# miR-513c-5p Suppression Aggravates Pyroptosis of Endothelial Cell in Deep Venous Thrombosis by Promoting Caspase-1

**DOI:** 10.3389/fcell.2022.838785

**Published:** 2022-04-04

**Authors:** Chu Chu, Bin Wang, Zhen Zhang, Wen Liu, Shangwen Sun, Gang Liang, Xiaoshan Zhang, Hongqiang An, Ran Wei, Xiaoxiao Zhu, Qiang Guo, Lin Zhao, Xiaoxiao Fu, Ke Xu, Xia Li

**Affiliations:** ^1^ Innovative Institute of Chinese Medicine and Pharmacy, Shandong University of Traditional Chinese Medicine, Jinan, China; ^2^ Department of Peripheral Vascular Disease, Affiliated Hospital of Shandong University of Traditional Chinese Medicine, Jinan, China; ^3^ School of Basic Medicine, Shandong First Medical University and Shandong Academy of Medical Sciences, Jinan, China; ^4^ The Key Laboratory of Cardiovascular Remodeling and Function Research, Chinese Ministry of Education, Chinese National Health Commission and Chinese Academy of Medical Sciences, The State and Shandong Province Joint Key Laboratory of Translational Cardiovascular Medicine, Department of Cardiology, Qilu Hospital of Shandong University, Jinan, China

**Keywords:** deep venous thrombosis, pyroptosis, vascular endothelial cell, miR-513c-5p, caspase-1

## Abstract

Deep vein thrombosis (DVT) is a common peripheral vascular disease. Secondary pulmonary embolism (PE) caused by DVT leads to substantial patient death. Inflammation has been suggested as a key factor in the pathophysiology of DVT, however, involvement of pyroptosis-related inflammatory factors in DVT formation remains unclear. Here, we proposed that post-transcriptional modification of caspase-1 might be a crucial trigger for enhanced pyroptosis in vascular endothelial cells (VECs), and consequently contributed to severer symptoms in DVT patients. In order to explore the involvement of pyroptosis in DVT, peripheral blood mononuclear cells were collected from 30 DVT patients, and compared with the healthy controls, we found caspase-1 was increased both in mRNA and protein levels. miRNA microarray analysis demonstrated that down-regulated miR-513c-5p was significantly negatively correlated with the expression of caspase-1. *In vitro* assays suggested that miR-513c-5p overexpression could ameliorate the expression of caspase-1, and thus decreased the production of cleaved gasdermin D (GSDMD) and interleukin (IL)-1β and IL-18 in VECs. The dual-luciferase reporter assay identified direct binding between miR-513c-5p and the 3′ untranslated region of caspase-1 encoding gene. The administration of miR-513c-5p mimics through tail vein injection or caspase-1 inhibitor (vx-765) by intraperitoneal injection remarkably decreased the volume of blood clots *in vivo*, whereas miR-513c-5p inhibitor aggravated thrombosis formation and this effect was dramatically weakened when treated in combination with vx-765. Collectively, these results revealed that the pyroptosis of VECs induced by decreased miR-513c-5p was involved in DVT progression and indicated a potential therapeutic strategy of targeting the miR-513c-5p/caspase-1/GSDMD signal axis for DVT management.

## Introduction

Deep vein thrombosis (DVT) is a clinically common disease with 1/1,000 incidence in the general population (Olaf and Cooney, 2017; Xu J. et al., 2020). DVT can occur in any deep vein of the pelvis, thigh, or calf, and when it falls off and deposits in pulmonary vessels that lead to pulmonary embolism, a substantial fraction of patients worldwide die of this disease each year (Appelen et al., 2017; Giordano et al., 2017; Zhang et al., 2019). In the later stage of DVT, 20∼50% patients develop post-thrombotic syndrome which seriously affects the quality of life (Rabinovich and Kahn, 2017). So far, the molecular biological mechanisms of DVT are unclear, and sensitive and specific biomarkers for the early diagnosis of DVT are currently lacking.

Pyroptosis is a kind of inflammatory programmed cell death mode between necrosis and apoptosis, which is characterized by pore formations in the cell membrane (Bergsbaken et al., 2009). Pyroptosis is programmed by two kinds of caspases, that is, caspase-1 and caspase-4/5/11. Caspase-1 is one of the most important members of the caspase family. It is activated by the complex of inflammasomes after sensing the pathogen signal. The activation of caspase-1 results in inflammation and pyroptosis (Cookson and Brennan, 2001). Activated caspase-1 promotes maturation of interleukin (IL)-1β and IL-18, induces the synthesis and release of other inflammatory cytokines, and amplifies local and systemic inflammatory responses (Yin et al., 2015). Furthermore, activated caspase-1 causes the cleavage of gasdermin D (GSDMD), and the cleaved product (N-terminal fragment) causes cell pyroptosis *via* forming open pores in the cell membrane and destroys cellular membranes (He et al., 2015; Shi et al., 2015). In addition, in some cases, GSDMD protein changes could also enhance pyroptosis and thus lead to inflammation (Shi et al., 2021; Wu et al., 2021). Inducing inflammatory reaction is a major feature of pyroptosis, which is different from other cell death modes such as apoptosis. Accumulating studies have identified that pyroptosis is involved in the occurrence and development of various diseases, including infectious diseases, diabetes, and cardiovascular diseases (Stienstra et al., 2010; Doitsh et al., 2014; Wang et al., 2020). However, the role of pyroptosis in DVT development has been barely explored.

MicroRNAs (miRNAs) are ∼22-nucleotide length endogenous noncoding RNAs (Diederichs and Haber, 2007). miRNAs could bind to its target gene directly through base complementary pairing, resulting in mRNA degradation or translational repression (Eulalio et al., 2008; Lozano et al., 2019). Extensive evidence has demonstrated the crucial role of miRNAs in various physiological and pathological processes such as cardiogenesis and tumorigenesis (Barwari et al., 2016; Li Q. et al., 2020). It has also been reported the involvement of miRNAs in DVT formation and development (Meng et al., 2015; Jiang et al., 2017a). Since the relationship between miRNAs and pyroptosis has been demonstrated recently (Xu Q. et al., 2020; Xu X. et al., 2020), we hypothesized that pyroptosis regulated by miRNAs is a possible mechanism for DVT development.

In this study, we identified up-regulated pyroptosis characterized by elevated expression of caspase-1 in PBMCs from DVT patients. miRNA microarray analysis revealed miR-513c-5p as the most significantly down-regulated miRNA in DVT patients, and miR-513c-5p expression could sensitively discriminate DVT from healthy controls evidenced using receiver operating characteristic (ROC) analysis. miR-513c-5p is a coding product of *MIR-513C*, which is localized on the human chromosome X. Little is known of miR-513c-5p function, except for some reports indicating that miR-513c-5p might play roles in the progression of thyroid cancer, neuroblastoma, or preeclampsia (Jia et al., 2020; Tong et al., 2021; Zhou et al., 2021). Our intensive functional and mechanistic investigation demonstrated that miR-513c-5p effectively inhibited caspase-1 expression by binding to its 3′ untranslated region (3′UTR). The knockdown of miR-513c-5p aggravated venous thrombosis and propagation by enhancing caspase-1-mediated pyroptosis, while the overexpression of miR-513c-5p could alleviate DVT formation *via* inhibiting caspase-1 expression. To our knowledge, this is the first investigation on miRNA-mediated pyroptosis dysregulation in DVT, and our findings may provide novel insights into the pathogenesis of DVT and contribute to identifying novel therapeutic strategies and potential diagnostic biomarkers of DVT.

## Materials and Methods

### Patients and Blood Specimen Collection

Between January 2018 and December 2019, 30 inpatient or outpatient DVT subjects at the Affiliated Hospital of Shandong University of Traditional Chinese Medicine were enrolled in this study. DVT was confirmed with color Doppler ultrasound and lower extremity angiography. All the patients had no history of chronic inflammatory diseases such as hypertension, diabetes, and arthritis (Supplementary Table S1). In addition, 30 age and gender matched healthy volunteers were included. Peripheral venous blood samples were obtained after overnight fasting for all subjects. PBMCs were isolated by Ficoll density-gradient centrifugation, and then total RNA and protein were extracted. This study was approved by the ethics committee of Shandong University of Traditional Chinese Medicine. The written informed consent was obtained from each participant.

### miRNA Microarray Analysis

miRNA microarray analysis was performed using total RNA extracted from PBMCs of six DVT patients and six control subjects by OE biotech Corporation (Shanghai, China). Data analysis, including obtaining digital signals, data normalization, and quality control were similar to our previous publication (Zhang et al., 2020a). We used the threshold values of fold change (DVT/control) > 5, *p* < 0.05 to select differentially expressed miRNAs. The microarray data have been deposited in the Gene Expression Omnibus (GEO) database (https://www.ncbi.nlm.nih.gov/geo) (GSE173461).

### Cell Culture and Transfection

Chemosynthetic mimics and inhibitor of miR-513c-5p, and respective negative controls (GenePharma, Shanghai, China) were transfected into HUVECs and PBMCs at a final oligonucleotide concentration of 100 nM with Lipofectamine™ 2000 (Invitrogen, Carlsbad, United States) for 24 h. The sequences of mimic negative controls (NC) were: 5′-UUC​UCC​GAA​CGU​GUC​ACG​UTT-3′ (sense), 5′-ACG​UGA​CAC​GUU​CGG​AGA​ATT-3′ (antisense); has-miR-513c-5p mimics were: 5′-UUC​UCA​AGG​UGU​CGU​UUA​U-3′ (sense), 5′-AAA​CGA​CAC​CUC​CUU​GAG​AAU​U-3′ (antisense); inhibitor negative control (INC) was: 5′-CAG​UAU​UUU​GUA​GUC​AA-3′; has-miR-513c-5p inhibitor was: 5′-AUA​AAC​GAC​ACC​UCC​UUG​AGA​A-3′.

### Dual-Luciferase Reporter Assay

HEK293T cells were co-transfected with caspase-1 wild type (WT) or mutant (Mut) 3′UTR inserted pGL3-3M-Luc plasmid (Promega, Madison, United States) and miR-513c-5p mimics (or negative control) for 24 h. The relative luciferase activity in the cells was examined using the Dual-Luciferase Reporter Assay Kit (Promega, United States).

### Real-Time Quantitative RT-PCR (qRT-PCR)

Total RNA from PBMCs of DVT patients and healthy controls or the experimental cell lines was extracted using the TRIzol Reagent (Invitrogen, Carlsbad, United States). miRNAs and mRNAs were reverse transcribed using the 1st Strand cDNA Synthesis Kit (Vazyme, Nanjing, China) and PrimeScript RT reagent Kit (Toyobo, Osaka, Japan), respectively, according to the manufacturer’s instructions. The relative expression of each RNA was quantified using SYBR Green (Invitrogen, Carlsbad, United States) based qRT-PCR assay on an Applied Biosystems 7500 instrument (Applied Biosystems, Foster, United States). U6 and GAPDH were used as internal controls for miRNA and mRNA expressional normalization, and relative RNA quantification was calculated *via* the comparative 2^−ΔΔCt^ method. For mRNA and miRNA expressional analysis, the PCR primer sequences used are shown in Supplementary Tables S2, S3. Unless otherwise stated, the amplification reaction for each sample was conducted in triplicate.

### Western Blot Analysis

Cells or tissue lysates and immunoblotting analysis were performed as described previously (Zhang Z. et al., 2020). Densitometric analysis was conducted using ImageJ software. The primary antibodies used for Western blot included caspase-1 (1:1,000, Abcam, United Kingdom, ab1872), GSDMD (1:1,000, Novus Biologicals, United States, NBP2-33422), caspase-4 (1:1,000, Abcam, United Kingdom, ab238124), caspase-11 (1:1,000, Abcam, United Kingdom, ab262899), and GAPDH (1:10,000, Abcam, United Kingdom, ab181603). All the experiments were repeated at least three times.

### Enzyme-Linked Immunosorbent assay (ELISA)

Human blood serum samples, culture supernatants of HUVECs, and the supernatant from grinding mouse vascular tissues were harvested and subjected to detect IL-1β and IL-18 using human or mice ELISA Kits (MultiSciences, Hangzhou, China), as we described previously (Zhang et al., 2020a). The assays were performed in triplicate.

### Immunofluorescence (IF)

HUVECs seeded on 48-well culture plates were transfected with NC, miR-513c-5p mimics, INC, and miR-513c-5p inhibitor when cells reached about 50% confluence. After 24 h post-transfection, the cells were fixed with 4% paraformaldehyde and subsequently blocked in 10% normal mouse serum for 30 min at room temperature. The cells were then incubated with the caspase-1 primary antibody (3.5 μg/ml; Proteintech, Wuhan, China) overnight at 4°C. The FITC-conjugated secondary antibody (20 μg/ml; BOSTER, Wuhan, China) incubation was followed to detect the corresponding antigen. The nucleus was counterstained with 4,6-diamidino-2-phenylindole (DAPI). Microscopy images were captured using the Olympus IX73 microscope. Each experiment was repeated at least three times.

### DVT Mouse Model and Treatment

Male C57BL/6J mice of 8-week-old were purchased from the SPF Biotechnology Company (Beijing, China). As we published before, the DVT mouse model was established by inferior vena cava (IVC) occlusion (Schonfelder et al., 2017). Mice were randomly divided into four treatment groups (*n* = 15 in each group), and treatments were given 30 min before IVC ligation (Sahu et al., 2017). 1) DVT + mimics NC group, DVT modeling surgery with 200 μl saline containing 5 nmol mimics NC *via* tail vein injection; 2) DVT + miR-513c-5p mimics group, DVT modeling surgery with 200 μl saline containing 5 nmol miR-513c-5p mimics *via* tail vein injection; 3) DVT + inhibitor INC group, DVT modeling surgery with 200 μl saline containing 10 nmol inhibitor INC *via* tail vein injection; 4) DVT + miR-513c-5p inhibitor group, DVT modeling surgery with 200 μl saline containing 10 nmol miR-513c-5p inhibitor *via* tail vein injection. Another set of mice (seven groups) was used for caspase-1 inhibitor (vx-765) administration: 1) Intact control group; 2) Sham surgery group; 3) DVT group; 4) DVT + vehicle treatment group, 5) DVT + vx-765 treatment group; 6) DVT + vehicle + miR-513c-5p inhibitor group; 7) DVT + vx-765+miR-513c-5p inhibitor group. vx-765 (Selleck Chemicals, Houston TX) was dissolved in 2% DMSO, 30% PEG300, and ddH_2_O. Based on previous studies (Li et al., 2019), vx-765 (50 mg/kg) or vehicle was injected intraperitoneally once daily for three consecutive days before miR-513c-5p inhibitor administration 48 h after treatment, Doppler ultrasound was performed to examine the IVC and then mice were euthanized for collecting 2 mm vascular tissue sections below the IVC ligation. Mice without thrombosis were excluded and the remaining mice were used for further analysis. For macrophage depletion studies, C57BL/6 mice were intravenously injected with 200 μl (5 mg/ml) clodronate-liposomes (Liposoma, Amsterdam, Netherlands) 1 day prior to and 1 day following IVC ligation (Zhao et al., 2019). All the animal work was approved by the Institutional Animal Care and Use Committee of Shandong First Medical University.

### Flow Cytometry

APC-conjugated antibody to mouse EGF-like module-containing mucin-like hormone receptor-like 1 (F4/80, 17-4801-82) and FITC-conjugated antibody to integrin subunit alpha M (CD11b, 53-0112-82) were from eBioscience (Ben Lomond, CA, United States). Flow cytometry was performed using the CytExpert software flow cytometer (Beckman Coulter Life Science, Indianapolis, Indiana, United States).

### Histological and Examination (H&E)

Similar as previously described (Zhang et al., 2020c). The stained tissues were scanned using an optical microscope (IX73, Olympus, Japan) and analyzed using Image-Toup View software.

### Murine Doppler Ultrasound

Similar as previously described (Zhou et al., 2017), the ultrasound images of the IVC and surrounding tissue were obtained using the Visualsonics Vevo 2100 system with a cardiovascular scan.

### Fluorescence *In Situ* Hybridization (FISH)

The FISH assay was conducted to visualize the expression and location of miR-513c-5p in mouse vascular tissue. Paraffin tissue sections of 5 μm thickness were hybridized using Cy3-labeled miR-513c-5p (Cy3-5′-ATAAACGACACCTCCTTGAGAA-3′) at 37°C overnight. The nucleus was counterstained with DAPI. Microscopy images were acquired with a laser scanning confocal microscopy (LSM780, ZEISS, Oberkochen, Germany). All the procedures were executed according to the manufacturer’s instructions (Genepharma, Shanghai, China).

### Statistical Analysis

Unless otherwise specified, all the experiments were repeated at least three times independently. The experimental results were presented as mean ± SD. The 2-tailed Student’s *t*-test was used to compare the data between any two groups through the normality and equal variance tests. If data for either normality or equal variance tests failed, nonparametric Mann–Whitney *U*-test was used. For multiple comparisons, one-way ANOVA on ranks with Bonferroni post hoc test was used. If data did not pass either test, then nonparametric Kruskal–Wallis test with Dunn post hoc test was used. Correlations were analyzed by Pearson correlation. The diagnostic value was evaluated using the ROC curve. Thrombus frequency was analyzed using Chi squared analysis. Data were plotted using GraphPad Prism 7.0 software. Statistical analyses were conducted using SPSS software (version 16.0). *p* < 0.05 was considered significant.

## Results

### Pyroptosis Is Up-Regulated in PBMCs of DVT Patients

**FIGURE 1:**
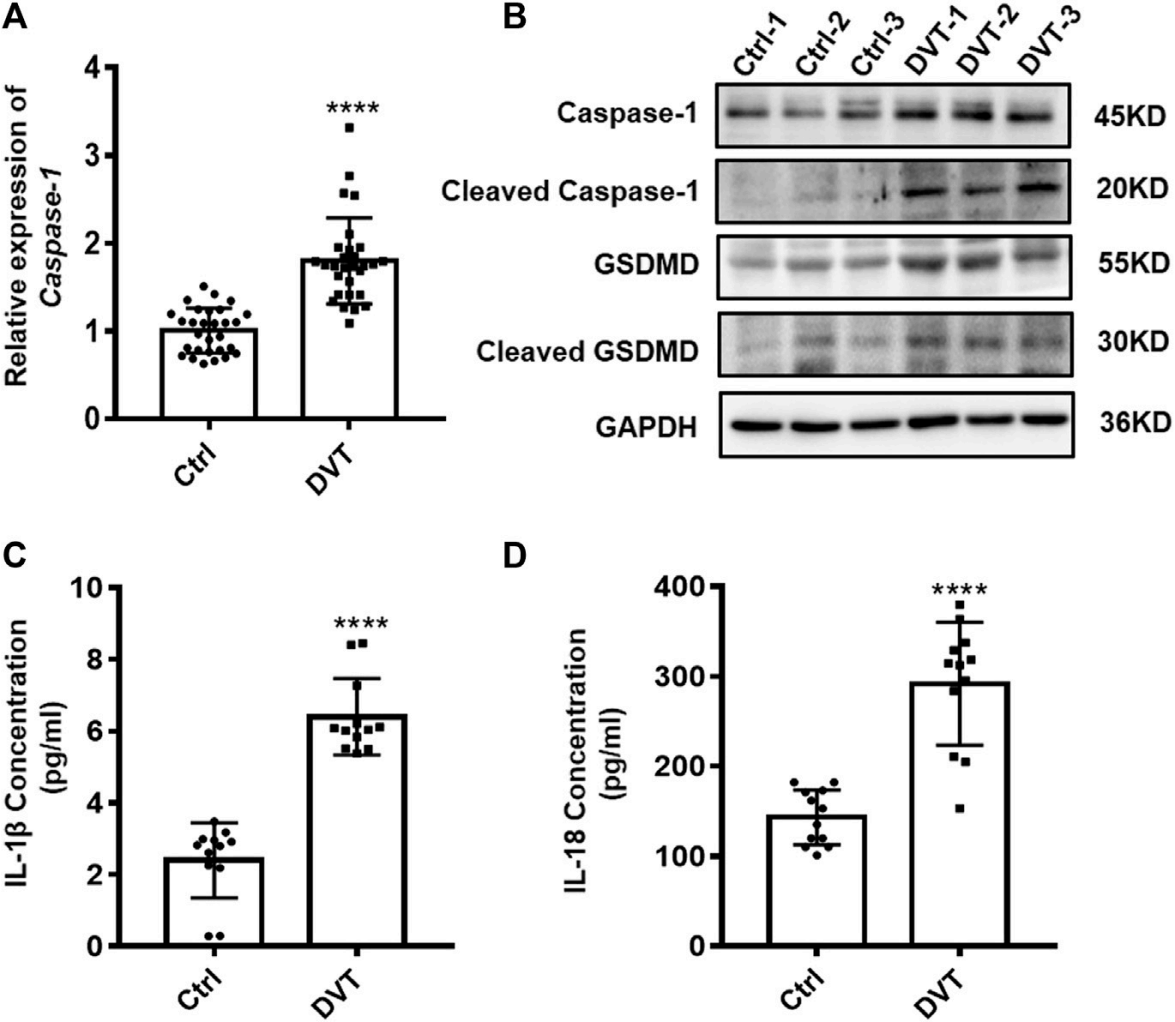
Expressions of caspase-1, GSDMD, IL-1β, and IL-18 are up-regulated in DVT patients. **(A)** mRNA level of caspase-1 in the PBMCs from 30 DVT patients and 30 controls was determined by qRT-PCR. **(B)** Caspase-1 and GSDMD protein levels were measured in PBMCs of DVT subjects and controls by Western blot. **(C,D)** Protein levels of IL-1β and IL-18 in the serum from 12 DVT patients and 12 controls were determined by ELISA. ****p < 0.0001.

Given that pyroptosis is dependent on the activation of caspase-1, which can cleave GSDMD and inactive precursor-pro-IL-1β and pro-IL-18 into their active forms and then induce pyroptosis, we first investigated whether there is variation in expression of caspase-1, GSDMD, IL-1β, and IL-18 under inferior vena cava flow-restricted conditions. qRT-PCR and Western blot analysis showed significant increases of the caspase-1 and GSDMD expressions in the PBMCs of DVT patients compared with those in normal controls (*p* < 0.05) (Figures 1A,B). Furthermore, the elevated protein levels of IL-1β and IL-18 were identified in the serum of DVT patients (*p* < 0.05) (Figures 1C,D). Taken together, these data provided evidence that the increased pyroptosis occurred in the development of DVT formation.

### miR-513c-5p Is Down-Regulated in DVT and Negatively Correlated with the level of Caspase-1

**FIGURE 2:**
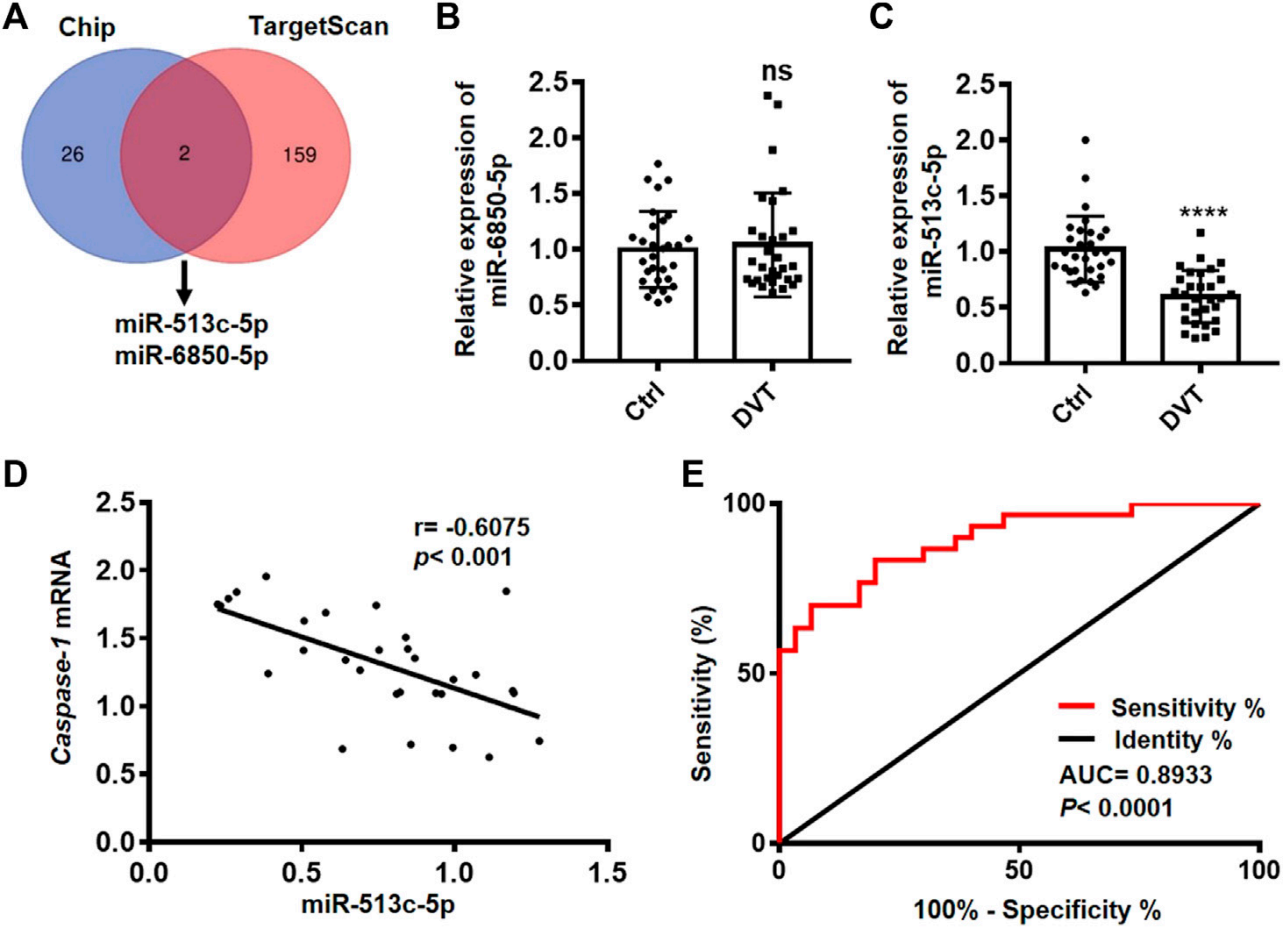
miR-513c-5p is down-regulated in DVT patients and negatively correlated with caspase-1. **(A)** The Venn diagram shows miR-513c-5p and miR-6850-5p predicted by TargetScan and Chip. **(B)** The expression level of miR-6850-5p in PBMCs from 30 DVT patients and 30 controls was measured by qRT-PCR. ns means no statistical difference. **(C)** The expression level of miR-513c-5p in PBMCs from 30 DVT patients and 30 controls was measured by qRT-PCR. **(D)** The correlation between miR-513c-5p and caspase-1 was analyzed using Pearson correction analysis (*n* = 30). **(E)** Diagnostic value of miR-513c-5p for DVT was evaluated by ROC curve. ****p < 0.0001.

To explore the regulatory miRNAs on pyroptosis in DVT, we examined the global miRNA profiles in the PBMCs of six DVT patients and identified 28 down-regulated miRNAs in the DVT group compared with the healthy controls. Since caspase-1 is the key initiator of pyroptosis, we took advantage of the target prediction database, TargetScan, to narrow down the potential miRNAs which might display a negative regulation on caspase-1 expression at transcriptional/post-transcriptional levels. These analyses suggested a high probability of interaction between caspase-1 and two differentially expressed miRNAs, miR-513c-5p, and miR-6850-5p (Figure 2A). We, then, further examined the expressions of miR-513c-5p and miR-6850-5p in the PBMCs of all 60 clinic samples. The data showed that the expression of miR-513c-5p was robustly decreased in DVT patients than that in normal controls, while no difference was detected for the expression of miR-6850-5p (Figures 2B,C). In addition, a significant negative correlation (*p* = 0.0004, *R* = −0.6075) was identified between miR-513c-5p and caspase-1 expression (Figure 2D), which further confirmed the involvement of miR-513c-5p in DVT. The subsequent ROC analysis demonstrated that miR-513c-5p level could sensitively distinguish DVT patients from healthy individuals (Figure 2E). These data indicated that decreased miR-513c-5p might enhance caspase-1 dependent pyroptosis in DVT. Pyroptosis is well characterized in myeloid lineage, especially macrophages, and whether pyroptosis changes in macrophage lead to DVT remains to be solved. In this study, we used clodronate-liposomes to eliminate macrophage in DVT mice, and measured whether the thrombosis was affected by macrophage depletion. The number of macrophages was significantly decreased upon clodronate-liposomes injection, which was detected using flow cytometry analysis (Supplementary Figure S1A). The thrombus size monitored by H&E staining was not altered by macrophage depletion (Supplementary Figure S1B). All together, we seek to study the role of pyroptosis in endothelial cells during DVT development.

### miR-513c-5p Negatively Regulates Caspase-1 Expression in VECs

**FIGURE 3:**
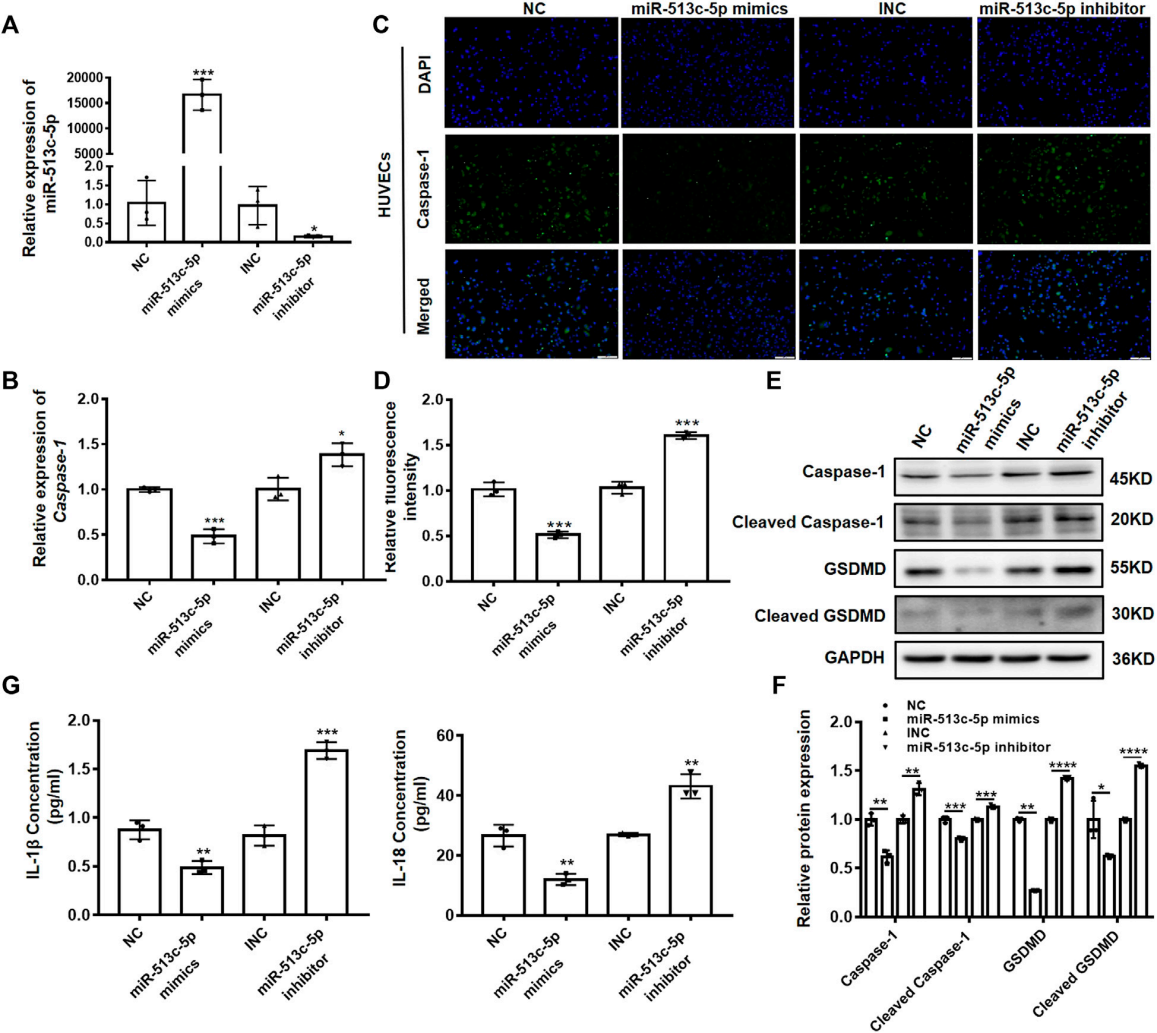
miR-513c-5p negatively regulates caspase-1 expression in HUVECs. **(A)** The expression of miR-513c-5p after NC, miR-513c-5p mimics, INC and miR-513c-5p inhibitor transfection was evaluated by qRT-PCR. **(B)** The expression level of caspase-1 mRNA after NC, miR-513c-5p mimics, INC and miR-513c-5p inhibitor transfection was detected by qRT-PCR. **(C,D)** Expression of caspase-1 in HUVECs were detected after transfection NC, miR-513c-5p mimics, INC, miR-513c-5p inhibitor by immunofluorescence staining (magnification, ×40). Scale bar = 200 μm. **(E,F)** Protein expression levels of caspase-1 and GSDMD were examined by Western blot. **(G)** The expression of IL-1β and IL-18 was detected by ELISA. *p < 0.05, **p < 0.01, and ***p < 0.001, ****p < 0.0001.

Endothelial cell injury and dysfunction are key factors in DVT formation. To verify the involvement of miR-513c-5p in the endothelial function, we altered the miR-513c-5p expression using its mimics and inhibitor in human umbilical vein endothelial cells (HUVECs), and then examined the expression of caspase-1. It was shown that miR-513c-5p expression was enhanced significantly after transfection of miR-513c-5p mimics, while the miR-513c-5p inhibitor exerted opposing effects (Figure 3A). In addition, we found that the mRNA expression of caspase-1 was decreased significantly by miR-513c-5p mimics while increased obviously by miR-513c-5p inhibitor, indicating that miR-513c-5p could inhibit the transcription of caspase-1 (Figure 3B). In addition, the immunofluorescence analysis results showed that the overexpression of miR-513c-5p robustly reduced the caspase-1 protein level in HUVECs, while the knockdown of miR-513c-5p exhibited the opposite effect (Figures 3C,D). Moreover, the data demonstrated that the protein level of GSDMD-N reduced synchronously by miR-513c-5p mimics, but this effect could be reversed by miR-513c-5p inhibitor, suggesting that decreased miR-513c-5p promoted pyroptosis mediated by caspase-1 (Figures 3E,F). Meanwhile, it was identified that protein levels of both IL-1β and IL-18 were decreased by miR-513c-5p mimics but increased by miR-513c-5p inhibitor, indicating the negative regulation of miR-513c-5p on inflammatory cytokines (Figure 3G). We also checked miR-513c-5p effects on caspase-1 in patient-derived PBMCs, and consistently found miR-513c-5p could significantly reduce the expression level of caspase-1, cleavage of GSDMD, and subsequently inhibited the yield of IL-1β and IL-18 (Supplementary Figures S2A–D).

In addition to caspase-1, the GSDMD protein could also be cleaved and activated by caspase-4/11, which makes it necessary to clarify whether the miR-513c-5p effect on DVT is solely dependent on caspase-1-mediated pyroptosis. As is known that mouse caspase-11 is the homolog of human caspase-4. To check the effect of miR-513c-5p on caspase-4, we perform qRT-PCR and Western blot assay upon miR-513c-5p overexpression or inhibition in HUVECs (Supplementary Figures S3A,B), and to check the effect of miR-513c-5p on caspase-11, we conducted the same assay using vascular tissue samples of DVT mice with miR-513c-5p mimics or inhibitor injection (Supplementary Figures S3C,D). The results showed that miR-513c-5p has no effect on caspase-4 or 11 on their mRNA or protein levels, which indicated miR-513c-5p suppression induced pyroptosis is not dependent on caspase-4/11.

### Caspase-1 Is a Direct Target of miR-513c-5p

**FIGURE 4:**
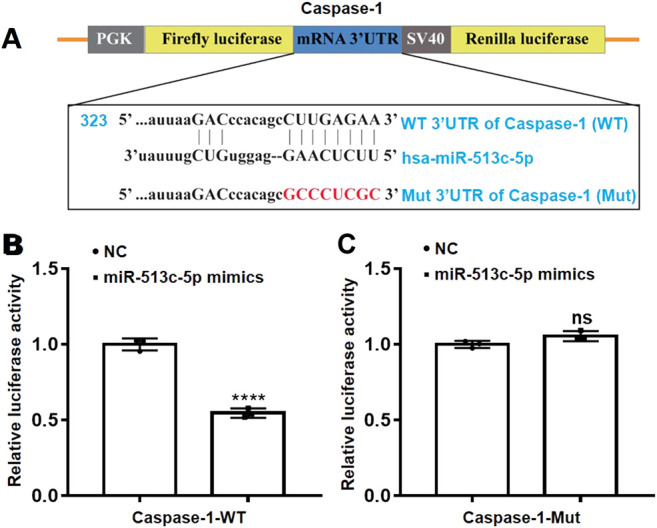
miR-513c-5p targets caspase-1 mRNA 3′UTR directly. **(A)** Schematic representation of caspase-1 mRNA 3′UTR demonstrating putative miRNA target site, luciferase activities of WT and Mut constructs. **(B,C)** The luciferase activity was determined by co-transfecting the vectors (caspase-1 3′UTR-WT or Mut) combined with NC, miR-513c-5p mimics into 293T cells, respectively. ****p < 0.0001.

To validate the *in silico* prediction and confirm the suppressive effect of miR-513c-5p on caspase-1 in post-transcriptional level, luciferase reporter assays were used with co-transfection of miR-513c-5p mimics and plasmids encoding WT or Mut 3′UTR of caspase-1 (Figure 4A). The presence of the miR-513c-5p mimics remarkably inhibited the activity of luciferase encoded within the WT caspase-1 plasmid. This was not observed in the presence of miR-513c-5p mimics and Mut caspase-1 (Figures 4B,C). These data confirmed that caspase-1 was the direct target of miR-513c-5p and down-regulated miR-513c-5p would increase caspase-1 mRNA and protein levels in DVT.

### Caspase-1 Inhibitor (vx-765) Inhibits Pyroptosis and DVT Formation *In Vivo*

**FIGURE 5:**
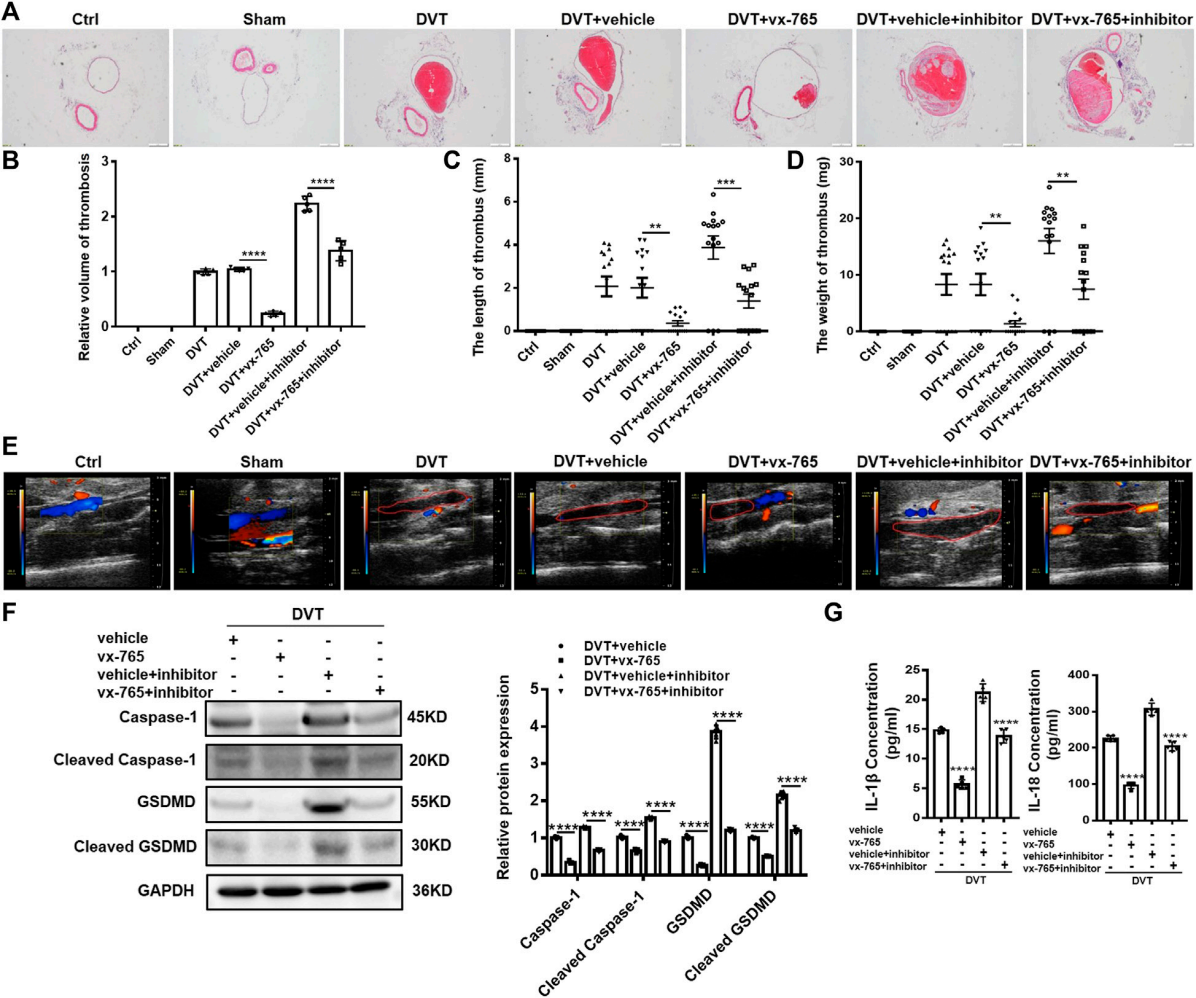
Caspase-1 inhibitor (vx-765) inhibits the formation of DVT *in vivo*. **(A,B)** H&E staining of serial cross sections of IVC from Ctrl, Sham, DVT, DVT+vehicle, DVT+vx-765, DVT+vehicle+miR-513c-5p inhibitor, DVT+vx-765+miR-513c-5p inhibitor at 48 h (magnification, ×40). Scale bars = 500 μm. **(C,D)** Thrombus length and weight at 48 h post-operation in the different treatment groups after the administration of caspase-1 inhibitor (vx-765) (*n* = 15 in each group). **(E)** Representative images of thrombi in each treatment group were detected by vascular ultrasound at 48 h post-operation. **(F)** Caspase-1 and GSDMD protein levels were determined by Western blot in vascular tissue of DVT animal group and vx-765-treated groups. **(G)** Expression of IL-1β and IL-18 in vascular tissue were detected by ELISA with vx-765 treated each group. **p < 0.01, ***p < 0.001, ****p < 0.0001.

Consistent with a previous report (Zhang et al., 2020a), 60% (9 out of 15 mice) of DVT models developed thrombosis in the current study. To elucidate the role of pyroptosis in DVT formation and development, we injected vx-765 into mice to inhibit the function of caspase-1. It was found that the thrombosis formation rate in mice decreased from 60% (9 out of 15 mice) to 40% (6 out of 15 mice) with the vx-765 treatment (Supplementary Figures S4A–C). Moreover, results from H&E staining and Doppler ultrasound showed that the size of blood clots in the vx-765 treated group dramatically diminished compared with those in the vehicle group (Figures 5A,B,E). Meanwhile, we found that both the length and weight of thrombus reduced nearly five folds in the vx-765 treatment group (Figures 5C,D). In addition, the protein levels of total and cleaved caspase-1 and N-terminal of GSDMD in the vascular tissue decreased significantly in the vx-765 treatment group (Figure 5F). In order to confirm the effects of DVT formation by miR-513c-5p-specific targeting of caspase-1, the DVT mice models were injected with vx-765 and miR-513c-5p inhibitor in combination. The results indicated that miR-513c-5p inhibitor enhanced caspase-1, GSDMD-N expression, and exhibited an aggravated thrombosis formation, and this effect was blunted by the co-administration of vx-765 (Figures 5A–F). Subsequently, we detected the concentrations of IL-1β and IL-18 in the vascular tissue and found that both cytokines were decreased by about 60 and 40%, respectively, after vx-765 treatment, and consistently, the miR-513c-5p inhibitor could exacerbate IL-1β and IL-18 production relieved by vx-765 (Figure 5G). These results indicated that pyroptosis mediated by miR-513c-5p targeted caspase-1 contributed to DVT pathogenesis.

### Pathogenic Role and Therapeutic Targeting miR-513c-5p in DVT *In Vivo*

**FIGURE 6:**
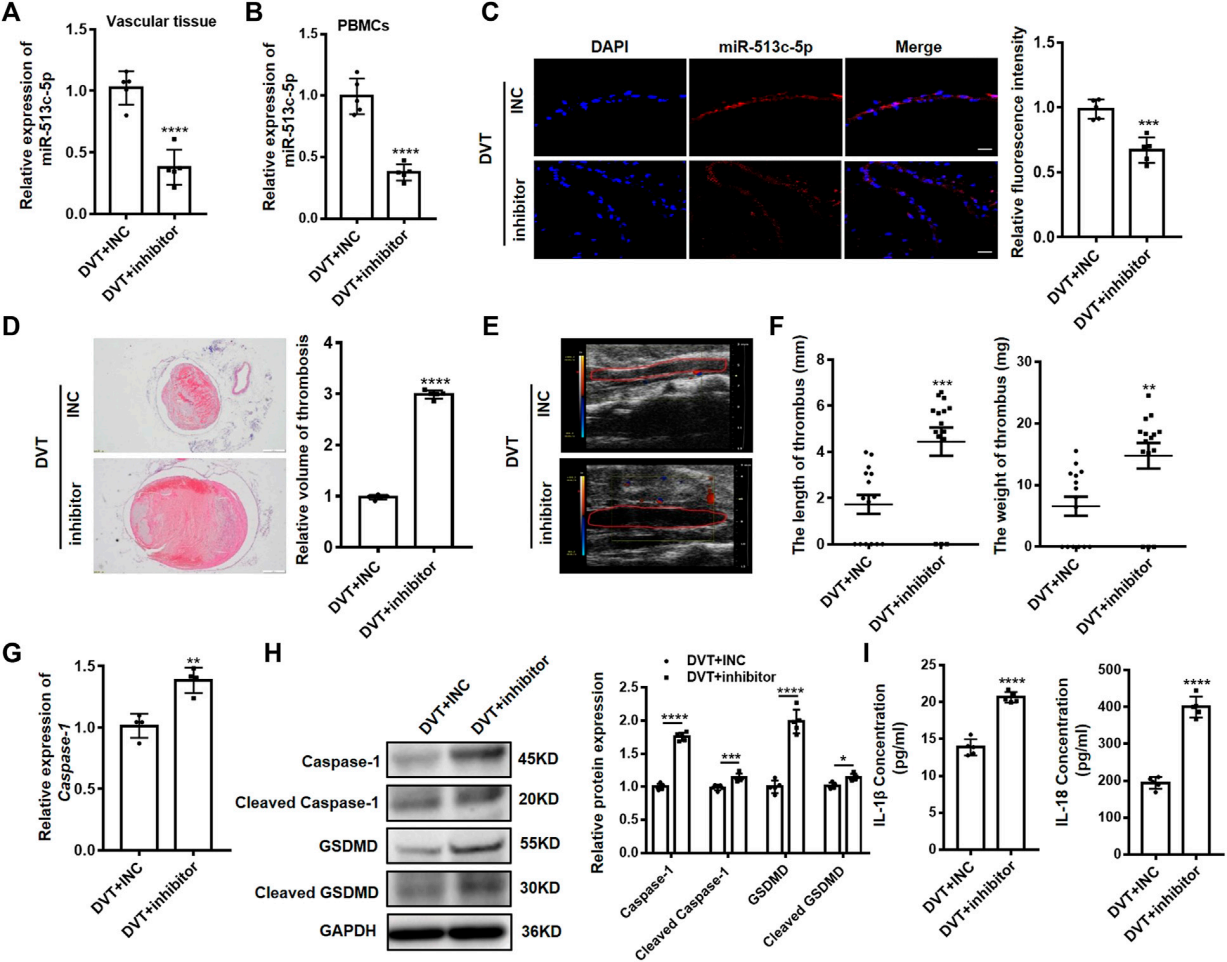
Knockdown of miR-513c-5p promotes pyroptosis of VECs and DVT formation by increasing caspase-1. **(A)** The expression level of miR-513c-5p in vascular tissue was detected by qRT-PCR in DVT mice treated with INC, miR-513c-5p inhibitor, respectively. **(B)** Expression of miR-513c-5p in PBMCs was detected by qRT-PCR in DVT mice treated with INC, miR-513c-5p inhibitor, respectively. **(C)** Confocal microscopy images of miR-513c-5p expression in vascular tissues (miR-513c-5p, red; DAPI, blue) (magnification, ×200). Scale bars = 100 μm. **(D) H**&**E** staining of serial cross sections of inferior vena cava (IVC) from DVT mice treated with INC, miR-513c-5p inhibitor at 48 h (magnification, ×40). Scale bars = 500 μm. **(E)** Representative images of thrombi in each treatment group were detected by vascular ultrasound at 48 h post-operation. **(F)** Thrombus length and weight at 48 h post-operation in the different treatment groups (*n* = 15 in each group). **(G)** Caspase-1 mRNA level was determined by qRT-PCR in vascular tissue of each treatment group. **(H)** Caspase-1 and GSDMD protein levels were determined by Western blot in vascular tissue of DVT mice treated with INC, miR-513c-5p inhibitor. **(I)** IL-1β and IL-18 protein levels in vascular tissue were determined by ELISA in INC, miR-513c-5p inhibitor treated DVT mice models, respectively. *p < 0.05, **p < 0.01, ***p < 0.001, ****p < 0.0001.

To further explore the pathogenic role of miR-513c-5p in DVT, a synthetic miR-513c-5p inhibitor was systemically administered to mice before undergoing the IVC ligation. We demonstrated the expected suppression of miR-513c-5p expression in IVC vascular tissue and PBMCs of DVT mice models (Figures 6A–C) and observed averagely larger blood clots in mice treated with the miR-513c-5p inhibitor than in the negative control group, by the H&E staining and Doppler ultrasound results (Figures 6D,E). In addition, the *ex vivo* thrombus length and weight were examined with 2.3-fold and 1.8-fold increases, respectively, in the miR-513c-5p inhibitor group (Figure 6F). Meanwhile, the mRNA expression of caspase-1 was increased by 1.4 folds in the miR-513c-5p inhibitor group (Figure 6G), and the protein levels of total and cleaved caspase-1, N-terminal of GSDMD, and IL-1β/IL-18 were increased significantly (Figures 6H,I).

**FIGURE 7:**
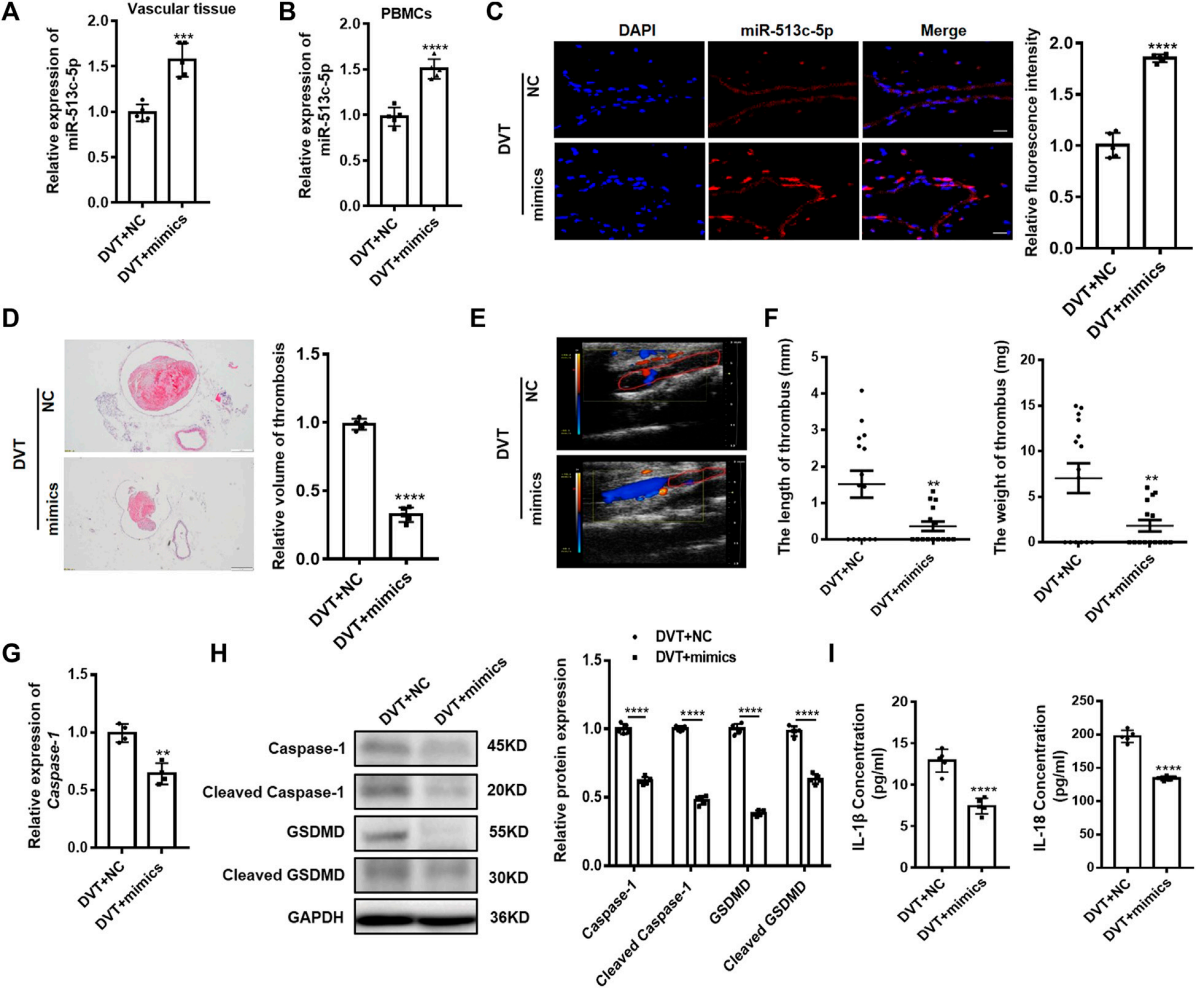
Overexpression of miR-513c-5p suppresses pyroptosis of VECs and DVT formation by inhibiting caspase-1. **(A)** Expression of miR-513c-5p in vascular tissue was detected by qRT-PCR in DVT mice treated with NC, miR-513c-5p mimics, respectively. **(B)** Relative expression of miR-513c-5p was measured by qRT-PCR in PBMCs of DVT each treatment group. **(C)** Confocal microscopy images of miR-513c-5p expression in vascular tissues (miR-513c-5p, red; DAPI, blue) (magnification, ×200). Scale bars = 100 μm. **(D)** H&E staining of serial cross sections of inferior vena cava (IVC) from DVT mice treated with NC, miR-513c-5p mimics at 48 h (magnification, ×40), respectively. Scale bars = 500 μm. **(E)** Representative images of thrombi in each treatment group were detected by vascular ultrasound at 48 h post-operation. **(F)** Thrombus length and weight at 48 h post-operation in DVT mice (*n* = 15) treated with NC, miR-513c-5p mimics. **(G)** Caspase-1 mRNA level was determined by qRT-PCR in vascular tissue of each treatment group. **(H)** Caspase-1 and GSDMD protein levels were determined by Western blot in vascular tissue of DVT each treatment group. **(I)** IL-1β and IL-18 protein levels in vascular tissue were determined by ELISA in DVT each treatment group. **p < 0.01, ***p < 0.001, and ****p < 0.0001.

We next tried to introduce miR-513c-5p mimics to compensate for the diminished miR-513c-5p in DVT mice. Upon successful increase of the miR-513c-5p expression in IVC vascular tissue and PBMCs of mice (Figures 7A–C), both H&E staining and Doppler ultrasound analysis showed that the volume of blood clots was decreased remarkably in the DVT mice (Figures 7D,E). The length and weight of blood clots were reduced by 4.6 folds and 4 folds separately in the miR-513c-5p mimics-treated mice (Figure 7F). The altered caspase-1/GSDMD/IL-1β/IL-18 signaling was restored by the miR-513c-5p mimics treatment accordingly (Figures 7G–I). Altogether, these data not only corroborated the pathogenic role of miR-513c-5p *in vivo*, but also suggested the therapeutic potential of chemosynthetic miR-513c-5p mimics in DVT.

## Discussion

**FIGURE 8:**
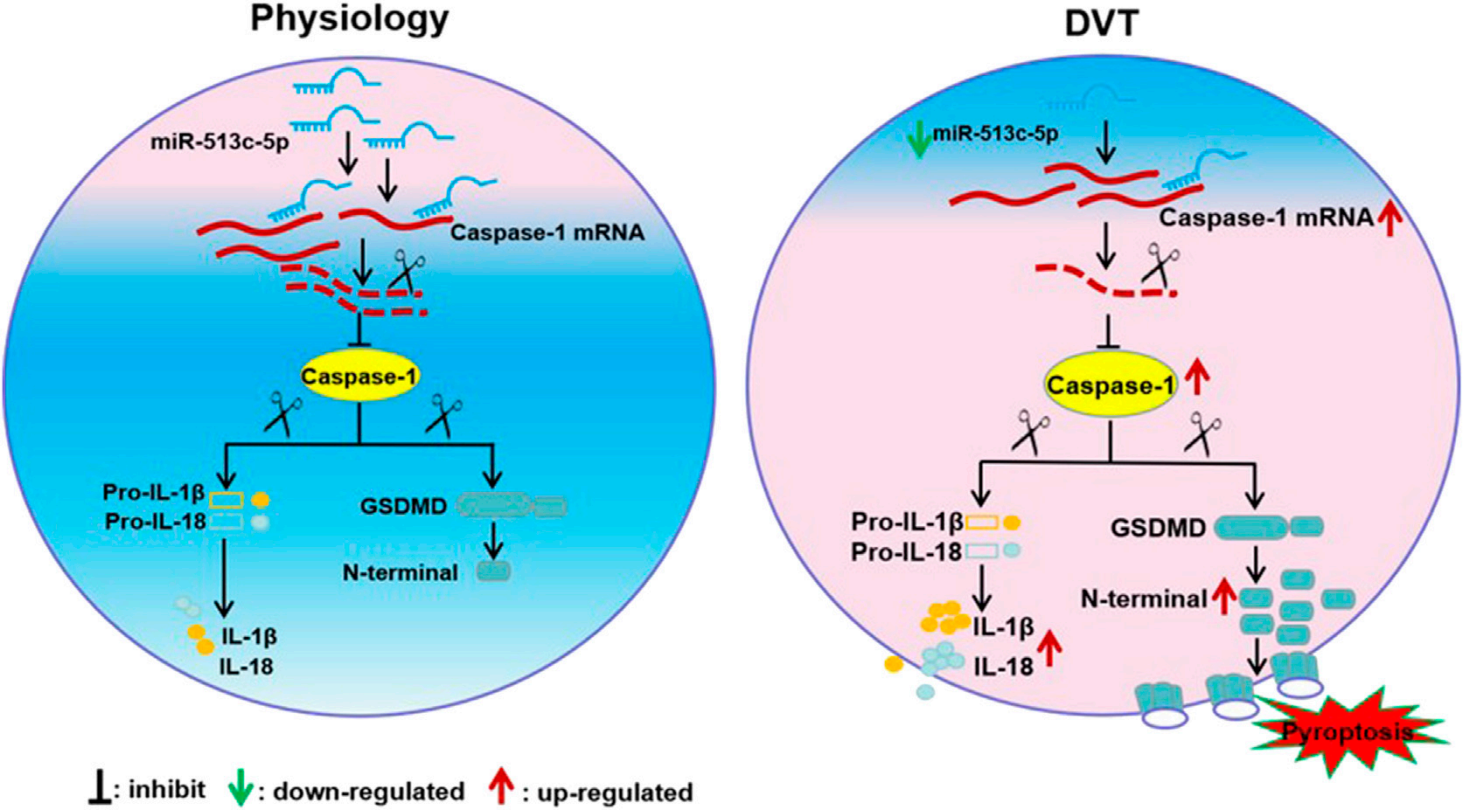
Schematic diagram of miR-513c-5p/caspase-1/GSDMD signal axis alteration in DVT pathogenesis. The down-regulation of miR-513c-5p in vascular endothelial cells relieved the inhibition of caspase-1 expression and resultant enhances of pyroptosis caused injury of endothelial cell, which lead to DVT formation.

In this study, we reported that the down-regulated miR-513c-5p contributed to the pathogenesis of DVT by promoting pyroptosis of VECs *via* enhancing caspase-1 expression. As far as we know, this is the first report on pyroptosis in DVT and the regulatory effect of miRNA on it. The regulatory network involving the miR-513c-5p/caspase-1 axis highlights a better understanding of the molecular mechanism on DVT formation and indicates potential for clinical diagnosis and therapeutic targeting of miR-513c-5p in DVT (Figure 8).

The miR-513c-5p is verified to be down-regulated in the process of DVT pathogenesis, however, the mechanism for its down-regulation has not been characterized here. There are possible reasons for the dysregulation of microRNA expression. First, miR-513c-5p down-regulation might be due to the genetic alterations, such as deletion or mutations, which caused inadequate expression or inhibited its maturation. Moreover, epigenetic changes such as methylation of the CpG islands of its promoter (Croce, 2009) and competing endogenous RNA regulatory network disorder probably lead to miR-513c-5p dysregulation (Tay et al., 2014). So it is of significance to in-depth revealing the mechanism for miR-513c-5p down-regulation, which might provide novel strategies for DVT interventions by targeting miR-513c-5p and pyroptosis.

We investigated the canonical pyroptosis pathway in DVT mediated by caspase-1. We are also aware of the limitations of our study that pyroptosis could also be mediated by non-canonical caspase-4/5 in humans and caspase-11 in murine (Broz, 2015; Xu et al., 2018). Once inflammasome is activated, caspase-1 and other non-canonical inflammasome caspases can cleave their substrate of GSDMD into N- and C- terminals to break the autoinhibitory interaction between them. The released N-terminal GSDMD then binds to the cellular lipid membrane to generate 12–14 nm pores, resulting in cell swelling and releasing inflammatory cytokines such as IL-1β and IL-18 (Kayagaki et al., 2011; Liu et al., 2016; Tsuchiya, 2020). This inflammatory phenomenon effectively distinguishes pyroptosis from apoptosis (Tsuchiya, 2020). Although a moderate pyroptosis is crucial for disrupting pathogen transmission into cells (Man et al., 2017), a widespread and uncontrolled pyroptosis can result in robust cell death, severe tissue damage, and is closely related to the pathogenesis of multiple diseases (Guo et al., 2019). Our data do not exclude the non-canonical pyroptosis in DVT pathogenesis, and the regulatory roles of miR-513c-5p on caspase-4/5 or caspase-11 warrant further investigation.

The roles of miRNAs in the formation and development of DVT are attracting more and more research attention recently. For example, down-regulated expression of miR-9-5p contributed to DVT formation by eliminating the inhibition of NF-κB signaling pathway (Ou et al., 2019). Up-regulated miR-483-3p in endothelial progenitor cells led to endothelial progenitor cells dysfunction *via* targeting serum response factor in DVT patients (Kong et al., 2016). The increased expression of miR-195-5p contributed to the development of DVT by enhancing the apoptosis of VECs (Jin et al., 2019). In addition, our previous studies found that decreased miR-338-5p was involved in the formation of DVT by enhancing IL-6 expression, while increased miR-374b-5p contributed to DVT *via* inhibiting IL-10 expression (Zhang et al., 2020a; Zhang et al., 2020c). These heterogeneous results highlighted the distinguished roles of cell-type-specific miRNAs and pointed to the potential temporal-evolved functions of miRNAs in the DVT development.

Similarly, increasing studies reported the regulatory effects of miRNAs on pyroptosis in various diseases. For instance, the overexpression of miR-135b suppressed pyroptosis and preserved cardiac function during myocardial infarction (Li A. et al., 2020). miR-214 inhibited the proliferation and migration of glioma cells through suppressing pyroptosis intermediated by caspase-1 (Jiang et al., 2017b). miR-590-3p suppressed pyroptosis in diabetic retinopathy by regulating the nicotinamide adenine dinucleotide phosphate oxidase four signaling pathway (Gu et al., 2019). In our study, we identified that decreased miR-513c-5p promoted endothelial cell pyroptosis mediated by caspase-1 which contributed to DVT formation. The miR-513c-5p/caspase-1 pathway might become novel diagnostic hallmarks and prospective therapeutic targets for DVT. Nevertheless, small RNA microarray and sequencing results uncovered that miR-513c-5p was up-regulated in patients with breast cancer and sex-cord stromal tumors (Chang et al., 2018; Muti et al., 2018). Therefore, future clinical application of miR-513c-5p mimics for DVT treatment might require careful caution.

## Data Availability

The datasets presented in this study can be found in online repositories. The names of the repository/repositories and accession number(s) can be found below: https://www.ncbi.nlm.nih.gov/geo/, GSE173461.
