# Supplementary material for: miR-513c-5p Suppression Aggravates Pyroptosis of Endothelial Cell in Deep Venous Thrombosis by Promoting Caspase-1
Source: Front Cell Dev Biol. 2022 Apr 4;10:838785. doi: 10.3389/fcell.2022.838785 (PMC9015708; doi:10.3389/fcell.2022.838785)
Supplement: Supplementary file 1 [file DataSheet1.docx]

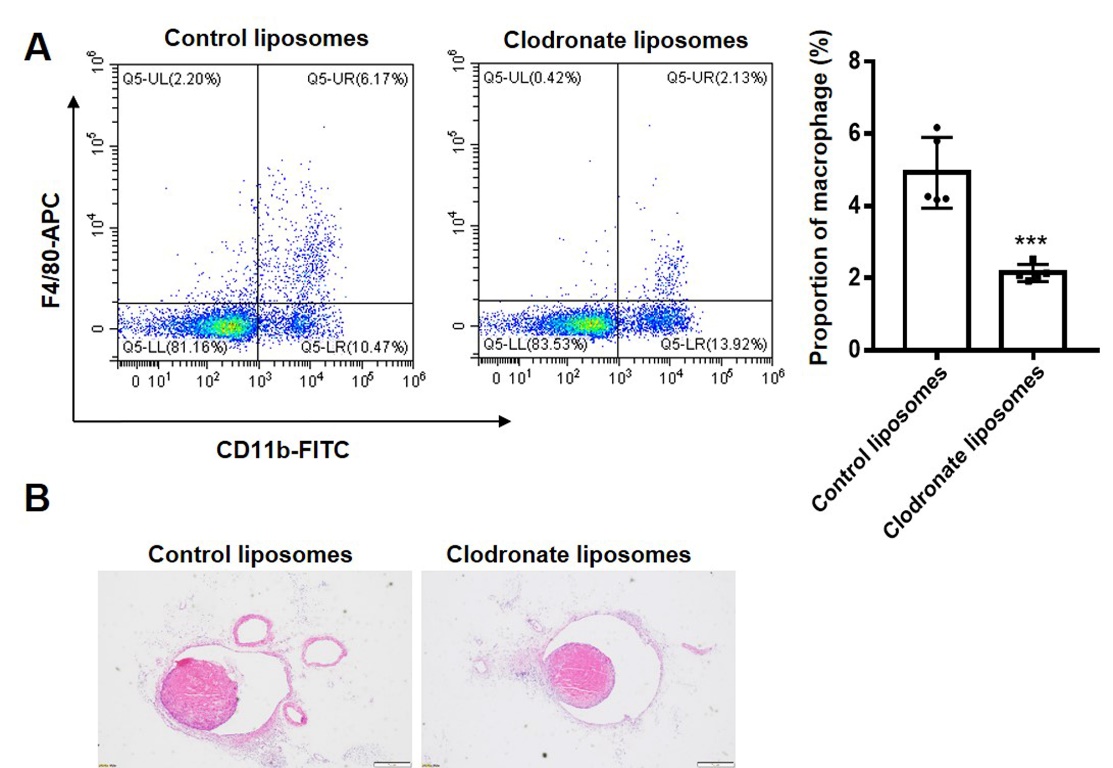


**Supplementary Figure 1 Effects of macrophage depletion on the thrombosis in DVT mice.**

**A** Mice were injected with control and clodronate liposomes, and macrophages from spleen were stained by the F4/80 and CD11b antibodies with the indicated labeling. The abundance of macrophages was detected by flow cytometry. **B** H&E staining of serial cross sections of inferior vena cava (IVC) from DVT mice treated with control and clodronate liposomes (magnification, ×40). Scale bars= 500 μm. ****P*< 0.001.


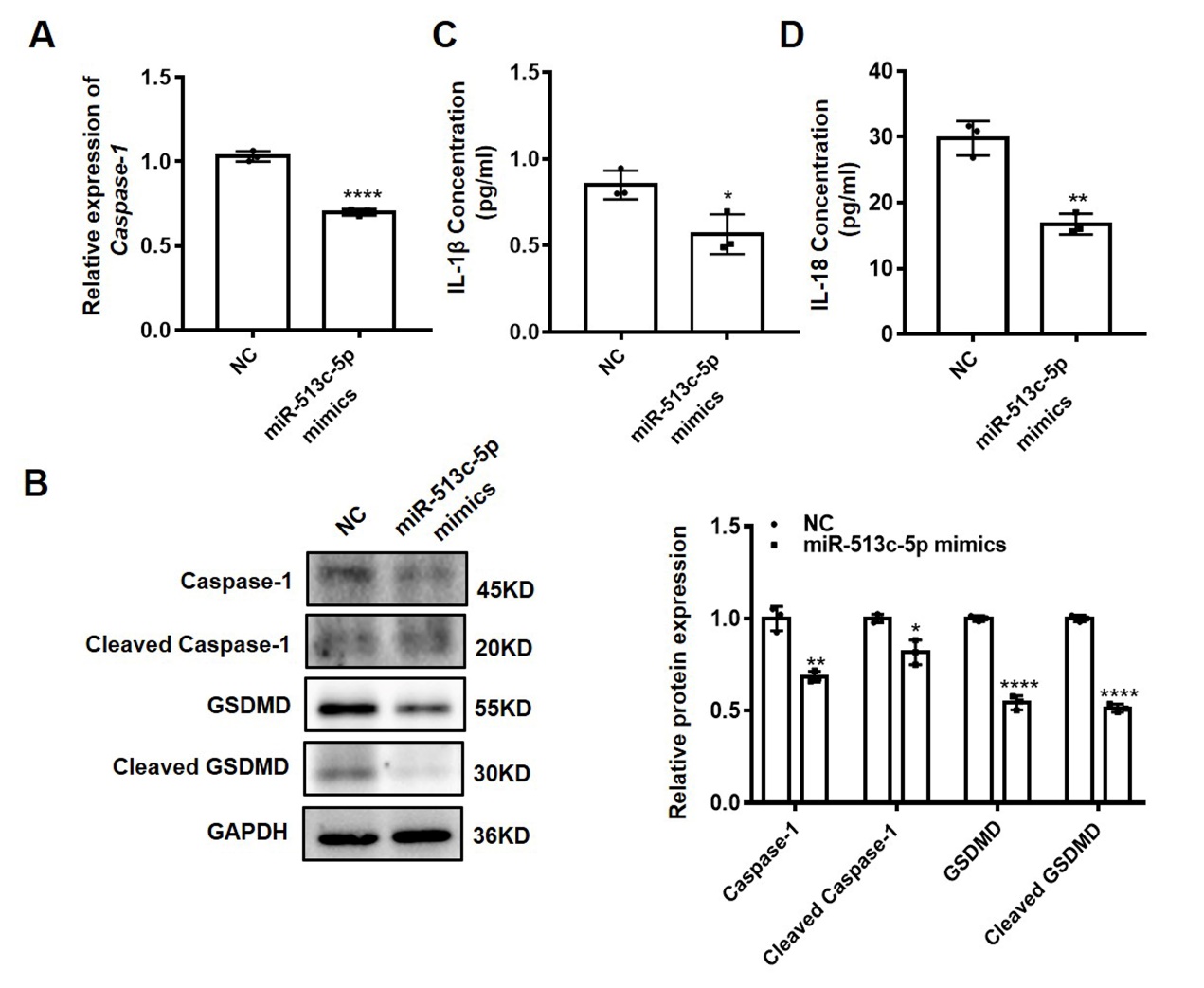


**Supplementary Figure 2 Regulatory effect of miR-513c-5p on Capsase-1/GSDMD in patient-derived PBMCs.**

**A** mRNA level of Caspase-1 was detected by qRT-PCR in patient-derived PBMCs. **B** Caspase-1 and GSDMD protein levels were examined by Western blot. **C, D** The expression of IL-1β and IL-18 was detected by ELISA from the culture medium of patient-derived PBMCs. **P*< 0.05, ***P*< 0.01, *****P*< 0.0001.


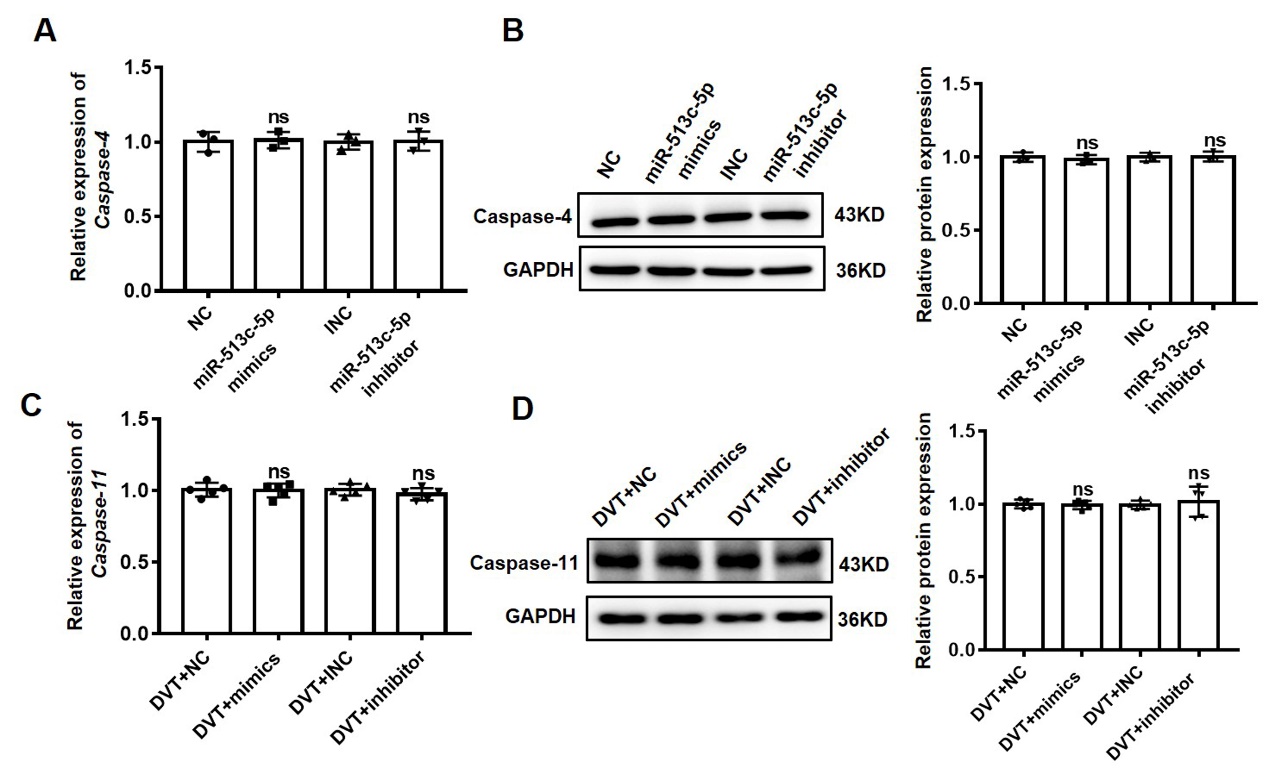


**Supplementary Figure 3 miR-513c-5p does not affect Caspase-4/11 expression.**

**A** mRNA expression of Caspase-4 was detected by qPCR in HUVECs. **B** Protein level of Caspase-4 was examined by Western blot in HUVECs. **C** mRNA expression of Caspase-11 in mice vascular tissue was detected by qRT-PCR. **D** Protein level of Caspase-11 in mice vascular tissue was detected by Western blot. ns: not significant.


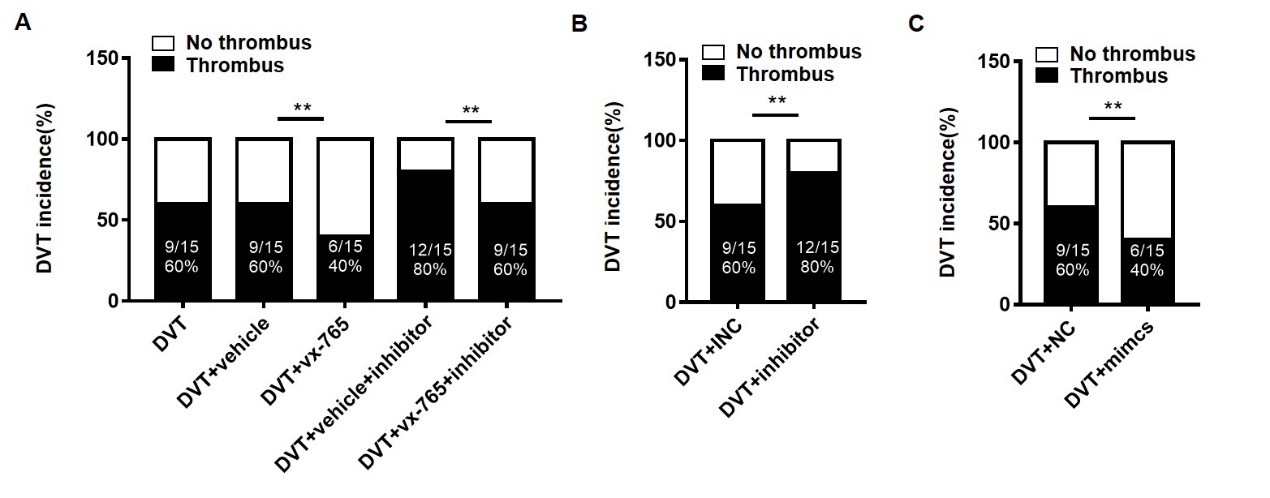
**Supplementary Figure 4** **Thrombus formation in different groups of DVT mice.**

**A** The thrombosis formation rate in mice with the vx-765 treatment. **B** The thrombosis formation rate in DVT mice treated with INC and miR-513c-5p inhibitor. **C** The thrombosis formation rate in DVT mice treated with NC and miR-513c-5p mimics. ***P*< 0.01.

**Supplementary Table 1 Baseline characteristics of DVT patients and healthy controls.**

| Characteristics | DVT  (n=30) | Control (n=30) | *P* vale |
| --- | --- | --- | --- |
| Age, years (mean± SD) | 56.7± 9.6 | 55.2± 8.9 | 0.5327 |
| Gender, females/males | 16/14 | 18/12 | 0.6023 |
| Weight (kg, mean± SD) | 60.2± 12.4 | 62.3± 15.6 | 0.5660 |
| BMI (kg/m^2^, mean± SD) | 24.2± 6.4 | 26.1± 6.6 | 0.2623 |
| Recent immobilization/Surgery | 0 | 0 | NA |
| Anti-coagulants or platelet-inhibitors | 0 | 0 | NA |
| Hormone | 0 | 0 | NA |
| Smoking | 0 | 0 | NA |
| Hypertension | 0 | 0 | NA |
| Diabetes mellitus | 0 | 0 | NA |
| Other chronic diseases | 0 | 0 | NA |

**Supplementary Table 2 List of** **polymerase chain reaction (PCR) primer sequences used in mRNA expression analysis.**

| **Gene** | **Species** | **Sequence (5'-3')** |
| --- | --- | --- |
| GAPDH | homo sapiens | Forward: ACAACTTTGGTATCGTGGAAGG  Reverse: GCCATCACGCCACAGTTTC |
| Caspase-1 | homo sapiens | Forward: CTCACTGCTTCGGACATGACT  Reverse: GCTGTCAGAGGTCTTGTGCT |
| Caspase-4 | Homo sapiens | Forward: AGTTTGACCATCTGCCTCCG  Reverse: GTCCAGACCCTCAAGTAGCTC |
| Actb | mus musculus | Forward: TCCTTCTTGGGTATGGAATCCTG  Reverse: TGCTAGGAGCCAGAGCAGTA |
| Caspase-4 | mus musculus | Forward: GGACTGACTGGGACCCTCAA  Reverse: GGCAAGACGTGTACGAGTGG |
| Caspase-11 | mus musculus | Forward: CCCCACATCACTTGTCCTACC  Reverse: CAAGGTTGCCCGATCAATGG |

**Supplementary Table 3 List of PCR primer sequences used in miRNA expression analysis.**

| **Gene** | **Sequence (5'-3')** |
| --- | --- |
| U6 | Forward: CAGCACATATACTAAAATTGGAACG  Reverse: ACGAATTTGCGTGTCATCC |
| miR-513c-5p | Forward: GCCGAGGCTGTGGAGGAACTC  Reverse: CAGTGCGTGTCGTGGAGT |
| miR-6850-5p | Forward: AAGTGCGGAACGTGGC  Reverse: TATGGTTGTTCACGACTGGTTCAC |
